# Supplementary material for: Reconciling Mining with the Conservation of Cave Biodiversity: A Quantitative Baseline to Help Establish Conservation Priorities
Source: PLoS One. 2016 Dec 20;11(12):e0168348. doi: 10.1371/journal.pone.0168348 (PMC5173368; doi:10.1371/journal.pone.0168348)
Supplement: S1 Dataset — (ZIP) [file pone.0168348.s002.zip › Taxa/Serra Norte/SN_2013/N4E/Lista N4E-25.pdf]

CAVIDADE N4E-0025

Classe	Ordem	Fam/Outros	Gên/Outros	Espécie	S-U	U-S	Zon.
Arachnida	Acari	Prostigmata		sp.	X		E
Arachnida	Amblypygi	Phrynidae	*Heterophrynus*	*longicornis*	X	1	E/P
Arachnida	Araneae	Ochyroceratidae	*Ochyrocera*	sp.	X	X	E
Arachnida	Araneae	Pholcidae		jovem	X		E
Arachnida	Araneae	Scytodidae	*Scytodes*	sp.	X		E
Arachnida	Araneae	Theraphosidae		sp.	X		E
Arachnida	Araneae	Theridiosomatidae	*Plato*	sp.1		X	E
Arachnida	Opiliones	Escadabiidae		sp.1	X		E
Arachnida	Pseudoscorpiones	Chernetidae	*Spelaeochernes*	sp.1		X	E
Diplopoda	Polydesmida	Pyrgodesmidae		jovem		X	E
Entognatha	Collembola	Sminthuroidea		sp.2	X	X	E
Insecta	Blattodea			jovem	X		E
Insecta	Coleoptera	Tenebrionidae		sp.	X		E
Insecta	Diptera	Psychodidae	*Psychodopygus*	*wellcomei*	X	X	E
Insecta	Hymenoptera	Formicidae	*Paratrechina*	sp.1		X	E
Insecta	Isoptera	Termitidae		sp.	X		E
Insecta	Orthoptera	Phalangopsidae	*Phalangopsis*	sp.		10	
Gastropoda	Pulmonata	Subulinidae	*Lamellaxis*	sp.	X		E
Malacostraca	Isopoda	Philosciidae		sp.	X		E
Amphibia	Anura			sp.	2	1	P
Mammalia	Chiroptera			sp.		3	P
